# Regulation of behavioral response to stress by microRNA-690

**DOI:** 10.1186/s13041-021-00728-3

**Published:** 2021-01-09

**Authors:** Jungyoung Park, Joonhee Lee, Koeul Choi, Hyo Jung Kang

**Affiliations:** Department of Life Science, Chung-Ang University, 84 Heukseok-ro, Dongjak-gu, Seoul, 06974 South Korea

**Keywords:** *Fkbp5*, MicroRNA, Medial prefrontal cortex, Stress

## Abstract

Psychiatric disorders are affected by genetic susceptibility and environmental adversities. Therefore, the regulation of gene expression under certain environments, such as stress, is a key issue in psychiatric disorders. MicroRNAs (miRNAs) have been implicated as post-transcriptional regulators of several biological processes, which can be differentially controlled through the targeting of multiple mRNAs. However, studies reporting the functions of miRNAs in relation to stress are lacking. In this study, we identified a significant increase in the expression of miRNA-690 (miR-690) in the medial prefrontal cortex (mPFC) of FK506-binding protein 51 knock-out (*Fkbp5* KO) mice. In addition, the expression pattern of miR-690 was similar to the sucrose preference of the same group in WT and *Fkbp5* KO mice. miR-690 was injected into the mPFC using a recombinant adeno-associated virus mediated gene delivery method. After recovery, miR-690 overexpressing mice were exposed to restraint stress for 2 weeks. In the sucrose preference test and forced swim test, the stressed miR-690 overexpressing mice showed higher sucrose preference and lower immobility time, respectively, than stressed mice injected with the control virus. In the novel object recognition test, the stressed miR-690 overexpressing mice interacted longer with the novel object than those injected with the control virus. These results showed that miR-690 might play a role in stress resilience and could provide new insights into the epigenetic regulation of stress-associated biological functions and diseases, such as depression and post-traumatic stress disorder.

Stress is a state in which homeostasis cannot be maintained, and both physical and psychological stressors have been reported to cause alterations in the endocrine system [1]. One of the major endocrine systems associated with stress response is the hypothalamic–pituitary–adrenal (HPA) axis, which comprises the hypothalamus, pituitary gland, and adrenal gland [2]. The hypothalamus releases corticotropin-releasing hormone upon exposure to stress, which stimulates the anterior pituitary gland to release adrenocorticotropic hormone (ACTH). ACTH stimulates the adrenal cortex to produce glucocorticoids [2]. FK506-binding protein 51 (FKBP5), a co-chaperone of heat shock protein 90 (Hsp90), acts as a regulator of the HPA axis. FKBP5 also interacts with Hsp90 to interfere with glucocorticoid receptor-mediated signaling [3, 4]. In our previous study, we found that *Fkbp5* knock-out (KO) mice exhibited significantly reduced depressive-like behaviors when exposed to stress, and transcriptomic analysis showed a distinct expression module associated with stress resilience in the medial prefrontal cortex (mPFC) [5]. The mPFC has been considered an important region associated with stress responses because it connects to several brain regions, such as the amygdala and hypothalamus, which regulate neuroendocrine and autonomic functions and mediate circuit-specific effects of stress on neuronal remodeling [6].

Over the past few years, epigenetics has been considered in the pathophysiology of many stress-related psychiatric disorders. Epigenetic modifications regulate gene expression without changing the original genetic code [7]. In particular, microRNAs (miRNAs) are highly expressed in the central nervous system and play important roles in the development of neural structures and the regulation of gene expression [8]. In addition, abnormal expression of miRNAs can lead to several neuropsychiatric disorders [9]. Therefore, elucidating the role of miRNAs can provide clues toward determining the mechanisms underlying these disorders. However, epigenetic regulation of miRNAs has not yet been fully investigated.

We conducted small RNA sequencing (RNA-seq) analysis to explore key miRNAs in the mPFC associated with stress, using *Fkbp5* KO mice, with wild-type (WT) mice as controls (Additional file 1). In the *Fkbp5* KO mice, which showed significantly lower *Fkbp5* expression levels (*P* < 0.0001, Additional file 3: Fig. S1), the expression levels of 41 miRNAs were altered, of which, 18 were upregulated and 23 were downregulated. Among the upregulated genes, the expression of microRNA-690 (miR-690) was significantly increased (fold change = 2.5128, adjusted *P* = 3.5 × 10^−3^) (Fig. 1a and Additional file 2: Table S1). Studies have reported that miR-690 plays roles in myeloid cell, osteogenic, and induced pluripotent stem cell differentiation and in the renin-angiotensin system [10–13]; however, the effects of miR-690 on stress response are unknown. We hypothesized that miR-690 could mediate the stress response. Therefore, to verify this hypothesis, miR-690 levels of *Fkbp5* KO mice were assessed when subjected to restraint stress for 3 weeks [5]. Restraint stress, a widely used animal model of stress, can alter neurotransmission and gene regulation in the short term and can cause neuronal structure modifications over the long term [14]. The effect of the restraint stress was confirmed by the sucrose preference test (SPT), and the miR-690 expression level was verified by quantitative PCR on total RNA extracted from the mPFC. The stressed WT (WT_ST) mice showed lower sucrose preference (*P* = 1.20 × 10^−3^) (Additional file 3: Fig. S2) and a lower level of miR-690 expression than the WT control (WT_CT) mice (*P* = 2.80 × 10^−3^) (Fig. 1b). On the other hand, stressed KO (KO_ST) mice, which showed significantly reduced depressive-like behavior (*P* = 1.10 × 10^–3^) (Additional file 3: Fig. S2), exhibited upregulated expression of miR-690 compared with WT_ST mice (*P* = 3.00 × 10^−4^) (Fig. 1b).

**Fig. 1:**
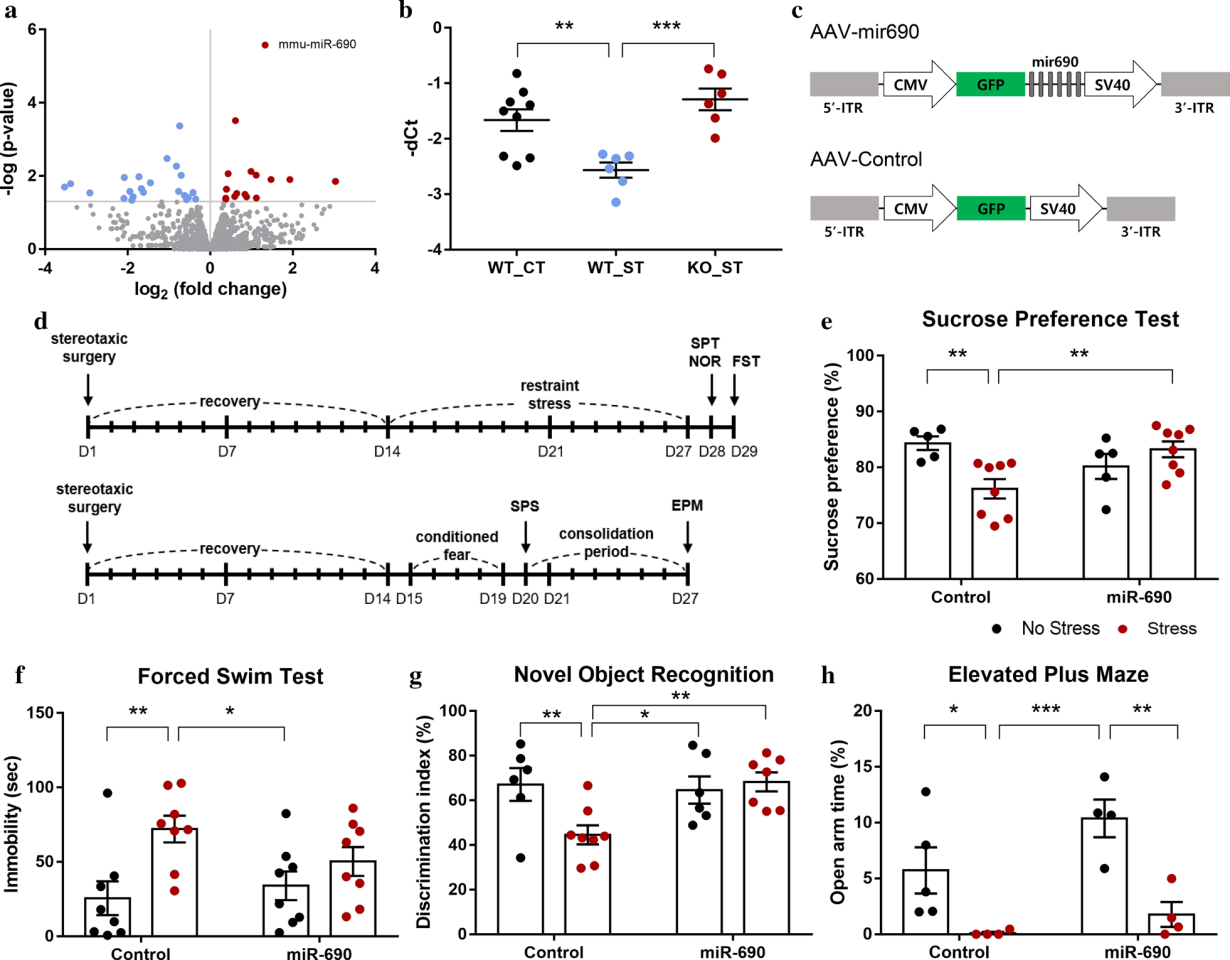
Overexpression of miR-690 in the mPFC led to stress-resilient behaviors following restraint stress in mice. **a** Volcano plot representing the differentially expressed miRNAs in the mPFC of *Fkbp5* KO mice, satisfying the criterion of *P* value < 0.05. Significantly altered miRNAs are indicated by blue (downregulated) and red (upregulated) dots. **b** The expression of mmu-miR-690 in the mPFC of mice subjected to the restraint stress. WT control mice (WT_CT, n = 9); stressed WT mice (WT_ST, *n* = 6); stressed *Fkbp5* KO mice (KO_ST, *n* = 6). One-way ANOVA (F [2, 18] = 10.65, *P* = 9.00 × 10^−4^); Fisher’s LSD (**P < 0.01, ***P < 0.001). **c** AAV-miR-690 and AAV-control vector design; *ITR* inverted terminal repeat, *CMV* cytomegalovirus; *GFP* green fluorescent protein, *SV40* simian virus 40. **d** Schematic timeline of experimental procedures and behavioral tests. Restraint stress (above) and conditioned fear stress combined with single-prolonged stress (CF + SPS) (below). **e–g** Influence of overexpression of miR-690 in the mPFC on mouse behavior following restraint stress. Results of sucrose preference test (**e**). Non-stressed control mice (*n* = 5); stressed control mice (*n* = 8); non-stressed miR-690 mice (*n* = 5); stressed miR-690 mice (*n* = 8). Two-way ANOVA (stress × virus, F [1, 22] = 10.39, *P* = 3.90 × 10^–3^); Fisher’s LSD (**P < 0.01). Results of forced swim test (**f**). Non-stressed control mice (*n* = 8); stressed control mice (*n* = 8); non-stressed miR-690 mice (*n* = 8); stressed miR-690 mice (*n* = 8). Two-way ANOVA (stress, F [1, 28] = 9.97, *P* = 3.80 × 10^–3^); Fisher’s LSD (*P < 0.05, **P < 0.01). Results of novel object recognition test (**g**). Non-stressed control mice (*n* = 6); stressed control mice (*n* = 8); non-stressed miR-690 mice (*n* = 6); stressed miR-690 mice (*n* = 7). Two-way ANOVA (stress × virus, F [1, 23] = 5.81, *P* = 2.44 × 10^−2^); Fisher’s LSD (*P < 0.05, **P < 0.01). **h** Effects of CF + SPS and miR-690 on elevated plus maze test. Non-stressed control mice (*n* = 5); stressed control mice (*n* = 4); non-stressed miR-690 mice (*n* = 4); stressed miR-690 mice (*n* = 4). Two-way ANOVA (stress, F [1, 13] = 20.45, *P* = 6.0 × 10^−4^); Fisher’s LSD (*P < 0.05, **P < 0.01, ***P < 0.001). In all data, black dots and red dots indicate non-stressed mice and stressed mice, respectively. Bars represent group mean and error bars represent SEM

We next infused either a control green fluorescent protein (GFP)-tagged recombinant adeno-associated virus (rAAV) or a viral construct containing miR-690 (rAAV-GFP-miR-690) into the pre-limbic cortices of the mPFC of mice to evaluate the effects of miR-690 on behavioral responses induced by chronic restraint stress (Fig. 1c and Additional file 3: Fig. S3). After 2 weeks of recovery, mice were subjected to restraint stress for 2 weeks. Effects of stress and the influence of miR-690 expression were verified by the SPT, forced swim test, and novel object recognition test (NOR), which are established models to assess mouse behavior (Fig. 1d). In the absence of stress, there was no significant influence on mice behavior by miR-690 expression itself; however, the effects appeared when the mice were exposed to stressful conditions. Mice injected with the control virus (rAAV-GFP) showed significantly lower sucrose preference (*P* = 3.50 × 10^−3^), increased total immobility time (*P* = 2.60 × 10^−3^) and decreased interaction rate with a novel object (*P* = 3.40 × 10^−3^) following restraint stress for 2 weeks; however, anhedonia, despair, and cognitive dysfunction caused by stress were blocked by overexpression of miR-690 in the mPFC (Fig. 1e–g and Additional file 3: Fig. S4). Furthermore, we tested whether the overexpression of miR-690 in the mPFC is associated with anxiety using another stress paradigm, conditioned fear stress combined with single-prolonged stress (CF + SPS). In the elevated plus maze test, overexpression of miR-690 did not prevent anxiety caused by CF + SPS (Fig. 1h). Although more detailed verification is required, miR-690 overexpression does not seem to have a significant effect on stress-induced anxiety. However, compared to the control virus, miR-690 overexpression tends to alleviate anxiety somewhat by overexpression itself, although not statistically significant.

In this study, we suggest that miR-690 could prevent depressive-like behaviors and cognitive dysfunction following exposure to restraint stress. Although further and more elaborate behavioral and molecular studies are necessary to identify the precise role of miR-690 in stress biology, our results demonstrated that miR-690 may be an epigenetic regulator of behavioral responses induced by chronic restraint stress. Because miRNAs can target multiple mRNAs and regulate their expression, determining the exact roles of miRNAs is essential. Therefore, additional studies associated with miR-690 and putative target genes will contribute toward providing therapeutic interventions for stress-related disorders.

## Supplementary Information

**Additional file 1.** Materials and methods.

**Additional file 2:** **Table S1.** Normalized count values of differentially expressed microRNAs (miRNAs) in *Fkbp5* knock-out (KO) mice

**Additional file 3:** **Figure S1.** *Fkbp5* expression level in the medial prefrontal cortex (mPFC) of *Fkbp5* knock-out (KO) mice. **Figure S2.** Effects of *Fkbp5* deletion and restraint stress on depressive-like behavior. **Figure S3.** The expression level of miR-690 after AAV-mediated gene transfer into the mPFC of mice. **Figure S4.** Novel and familiar objects interaction time in the novel object recognition test.

## Data Availability

The small RNA-seq raw data reported in this paper have been submitted to the Gene Expression Omnibus repository under the accession number (GSE139502).

## Abbreviations

ACTH: Adrenocorticotropic hormone
CF + SPS: Conditioned fear stress combined with single-prolonged stress
Fkbp5: FK506-binding protein 5
GFP: Green fluorescent protein
HPA axis: Hypothalamic–pituitary–adrenal axis
Hsp90: Heat shock protein 90
KO: Knock-out
KO_ST: Stressed KO
miRNA: MicroRNA
mPFC: Medial prefrontal cortex
NOR: Novel object recognition
rAAV: Recombinant adeno-associated virus
RNA-seq: RNA sequencing
SPT: Sucrose preference test
WT: Wild-type
WT_CT: WT control
WT_ST: Stressed WT
