# Supplementary material for: Regulation of behavioral response to stress by microRNA-690
Source: Mol Brain. 2021 Jan 9;14:7. doi: 10.1186/s13041-021-00728-3 (PMC7797085; doi:10.1186/s13041-021-00728-3)
Supplement: Supplementary file 1 — Additional file 1. Materials and methods. [file 13041_2021_728_MOESM1_ESM.docx]

**Additional File 1**

Regulation of behavioral response to stress by microRNA-690

Jungyoung Park^1^, Joonhee Lee^1^, Koeul Choi^1^, and Hyo Jung Kang*^1^

^1^Department of Life Science, Chung-Ang University, Seoul 06974, South Korea

**Materials and Methods**

**Animals**

We obtained strains of *Fkbp5* KO (JAC stock #017989) mice from the Jackson Laboratory (Bar Harbor, ME), and stabilized the lines by backcrossing them with C57BL/6 mice. WT and *Fkbp5* KO mice were used for small RNA sequencing and validation of miR-690 expression. C57BL/6 male mice were obtained from Orient Bio (Seongnam, Korea) and were used to confirm the role of miR-690. Different cohorts were used for each behavioral test. Mice were housed under standard laboratory conditions (18-26℃, 45-55% humidity) in a 12 h-light/dark cycle (light from 07:30 to 19:30 hours). Mice were provided adequate food and water at all times, except during the restraint stress and behavioral tests. All animal care and experimental protocols were in accordance with the Chung-Ang University Animal Research Ethics Committee (Seoul, Korea).

**Small RNA-seq in *Fkbp5* KO mice**

Eleven-week-old WT (n = 3) and *Fkbp5* KO mice (n = 4) were used for small RNA sequencing. Total RNA was isolated from the mPFC using the miRNeasy Mini Kit (Qiagen, Hilden, Germany), and the RNeasy MinElute Cleanup kit (Qiagen) was used for miRNA enrichment. Small RNA cDNA libraries were generated with the NEXTflex Small RNA-Seq Kit v3 (PerkinElmer, Waltham, MA) according to the manufacturer’s instructions. Adapters were ligated to RNAs in each sample, and cDNAs were amplified by PCR. Sizes were selected using 6% TBE gel, and only bands ranging from 140 to 160 base pairs in size were analyzed. After gel purification, the library was paired end sequenced on a HiSeq 2500 (Illumina, San Diego, CA). FastQC (v0.11.5) was used for quality control of the raw RNA sequencing data, and adapter sequence trimming of the raw reads was performed using cutadapt v1.11. Raw reads were aligned to the reference genome using Bowtie v1.1.2 (http://bowtie bio.sourceforge.net/), and aligned reads were quantified using HTSeq (version 0.6.1p1). The R package edgeR (v3.10.2) was used to define differentially expressed genes. Fisher's exact test was used to calculate the *P*-values, which were corrected through the Benjamini-Hochberg method using the p.adjust function in the R package.

**miRNA PCR in *Fkbp5* KO mice**

Eight-week-old male WT and *Fkbp5* KO mice were used in this study and were divided into three groups: WT_CT (n = 9), WT_ST (n = 6), and KO_ST (n = 6). Mice were subjected to chronic restraint stress using restraint bags for 6 h a day for 3 weeks, with limited water and food. Mice in the control group were maintained under the same conditions but were not subjected to the chronic restraint stress. The SPT was performed to confirm the effect of restraint stress, following which the mice were sacrificed by cervical dislocation and the mPFC was dissected. Total RNA was isolated from the mPFC using a miRNeasy Mini Kit (Qiagen), and miRNA enrichment was performed using a RNeasy MinElute Cleanup kit (Qiagen) according to the manufacturer’s instructions. miRNAs (20 ng) were used for cDNA synthesis using TaqMan universal master mix (Thermo Fisher Scientific, Waltham, MA) and TaqMan RT primers (Thermo Fisher Scientific, miR-690, 001677; U6, 001973; miR-16, 000391). Quantitative PCR was conducted on a QuantStudio™ 6 Flex Real-Time PCR System (Thermo Fisher Scientific), and the Ct values were normalized using U6 and miR-16 as internal controls.

**Virus**

The AAV-control (pAAV_DJ_-CMV-GFP-MCS-SV40) and AAV-miR-690 vectors were purchased from ABM (ABM Industries, Vancouver, Canada). In the AAV-miR-690 vector, the entire miR-690 sequence was inserted in the multiple cloning site of the AAV-control vector. The complete sequence of miR-690 was as follows (Accession no. MI0004658); TGTGTTTTTGTGGAGCTAATTGGCTGTATTAAAGTGCTAGTAAGAAACATTCTCCTCCAGCTGGAGAGATGGCTCAGCTGTTAAAGGCTAGGCTCACAACCAAAATATA. The AAV was packaged in the KIST virus facility (http://virus.kist.re.kr). The titer of the AAV-control was 1.64 × 10^12^ GC/mL and that of AAV-miR-690 was 6.95 × 10^11^ GC/mL.

**Stereotaxic surgery**

Mice were anesthetized with a mixture of ketamine (87.5 mg/kg, i.p.) and xylazine (12.5 mg/kg, i.p.). When the mice lost consciousness, the front teeth were hooked and the skull was fixed with an ear bar. An anterior-posterior incision was made approximately 3 cm from the midline of the scalp of each mouse. Considering the bregma as reference, the coordinates were 15° (angle), +1.7 mm (anterior/posterior), +0.75 mm (medial/lateral), and -2.5 mm (dorsal/ventral) and the skulls were drilled to fit the coordinates. A Hamilton syringe (Hamilton, Reno, NV) was gradually lowered to the calculated mark, and a total of 1 µL of the virus was injected bilaterally over 5 min. After injecting the virus, the mice were remained for 5 min, following which they were returned to their cages and allowed to recover for a period of 2 weeks.

**Restraint stress**

Mice were placed in 50-mL conical tubes (SPL Life Sciences, Pocheon, Korea) and subjected to restraint stress for 6 h (from 10:00 am to 4:00 pm) per day for 14 days. When the mice were exposed to stressors, water and food were restricted; however, holes were drilled in the conical tubes to facilitate breathing. After the experiment, the mice were immediately returned to their cages and provided food and water *ad libitum*. Control group mice were maintained under the same conditions but were not subjected to the restraint stress.

**Conditioned fear stress combined with single-prolonged stress**

Mice were habituated for 60 s in the fear conditioning box (250 × 250 × 250 mm) (Panlab, Barcelona, Spain) and a light was turned on in the chamber [1]. After 10 s, mice were exposed to inescapable 1 mA foot shock for 2 s, which was repeated for 5 consecutive days. On day 6, mice suffered single-prolonged stress (SPS). Mice were subjected to restraint stress for 2 h in 50 ml conical tubes (SPL Life Sciences) and immediately followed by a group forced swim for 20 m (23-25 °C). After 15 m of recuperation, mice were moved to a sperate chamber and exposed to diethly ether (Duksan, Seoul, Korea) until the loss of consciousness. Ether exposure was conducted by placing cotton balls soaked with ether and placed into the chamber.

**Sucrose preference test**

The mice were placed in a single cage before the test, acclimated to 1% sucrose water for 24 h, and then deprived of water for 24 h. Subsequently, the mice were provided with two identical bottles: one with tap water (normal water) and the other with 1% sucrose water. Mice had *ad libitum* access to the cage, and liquid intake was measured for 16 h. Sucrose preference (%) was measured as follows: sucrose consumption (g)/total liquid intake (g) × 100.

**Forced swim test**

The mice were habituated for 1 h in a behavioral test room before the actual test and were forced to swim for 6 min in a cylinder (5 cm × 30 cm) filled with tap water (23-25°C, up to a height of 20 cm from the base). The tests were videotaped, and only the last 4 min was considered the total immobility value.

**Novel object recognition**

This test comprised training and test sessions. First, the mice were adapted to a behavioral test room for 1 h under a red light (5 lux). During the training session, the mice were allowed to run freely for 5 min in an open-field box (40 cm × 40 cm × 40 cm) and then explore the objects located in the corner of the box for 5 min. After 24 h, a test session was performed, wherein one of the training objects was replaced with a novel item, and the distinctive interaction of the mice with the two objects was observed for 5 min. All tests were video-recorded, and measurements were performed manually when the mice interacted with the objects. Discrimination index (%) was measured as follows: total time spent with the novel object (s)/(total time spent with the novel object (s) + total time spent with the familiar object (s)) × 100.

**Elevated plus maze**

Before the test, mice were habituated for 1 h in the test room under a red light (5 lux). Mice were placed at the junction of the four arms of the elevated plus maze. Mice were allowed to move around freely for 5 m in the maze and the video-tracking Smart 3.0 software (Panlab) identified their movement in real time. A state of entry was defined as a placing of four paws on the open arms. Open arm time (OA time) (%) was measured as follows: time spent in the open arms / (time spent in the open arms + closed arms) x 100.

**Statistical analysis**

All results were illustrated using GraphPad Prism v7.0 (GraphPad Software, San Diego, CA), and the unpaired *t*-test, one-way analysis of variance (ANOVA), and two-way ANOVA were used to express differences between means. Data are represented as means ± standard errors of the mean, and *P*-values < 0.05 were considered significant. Significant outliers were calculated with the extreme Studentized deviate method at a significance level of 0.05 (two-sided).

**References**

1. Wang H, Zuo D, He B, Qiao F, Zhao M, Wu Y: Conditioned fear stress combined with single-prolonged stress: a new PTSD mouse model. Neuroscience research. 2012;73:142-152.
