# Supplementary material for: Regulation of behavioral response to stress by microRNA-690
Source: Mol Brain. 2021 Jan 9;14:7. doi: 10.1186/s13041-021-00728-3 (PMC7797085; doi:10.1186/s13041-021-00728-3)
Supplement: Supplementary file 3 — Additional file 3: Figure S1. Fkbp5 expression level in the medial prefrontal cortex (mPFC) of Fkbp5 knock-out (KO) mice. Figure S2. Effects of Fkbp5 deletion and restraint stress on depressive-like behavior. Figure S3. The expression level of miR-690 after AAV-mediated gene transfer into the mPFC of mice. Figure S4. Novel and familiar objects interaction time in the novel object recognition test. [file 13041_2021_728_MOESM3_ESM.docx]

**
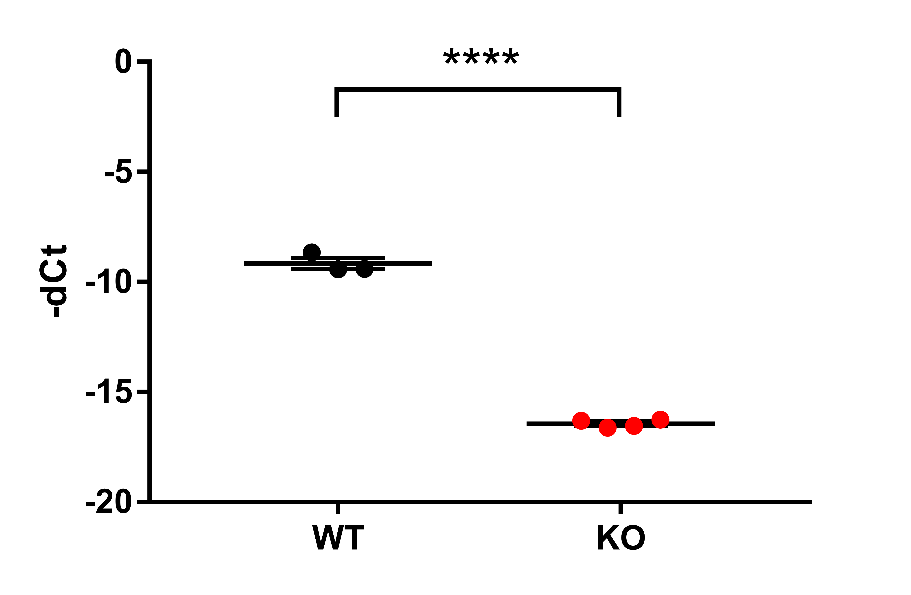
**

**Figure S1.** *Fkbp5* expression level in the medial prefrontal cortex (mPFC) of *Fkbp5* knock-out (KO) mice (fold change = 0.006). WT, n = 3; KO, n = 4. The data were analyzed by unpaired *t*-test, and error bars represent standard error of the mean (SEM). **** *P* < 0.0001


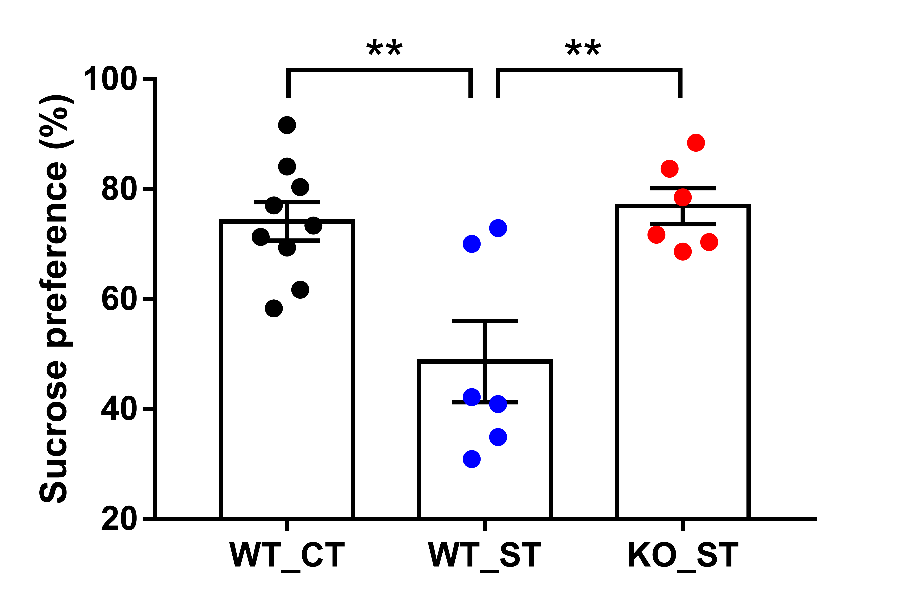


**Figure S2.** Effects of *Fkbp5* deletion and restraint stress on depressive-like behavior confirmed by sucrose preference test (SPT). WT control mice (WT_CT, n = 9); stressed WT mice (WT_ST, n = 6); stressed *Fkbp5* KO mice (KO_ST, n = 6). One-way ANOVA (F [2,18] = 9.618, *P* = 1.40 × 10^-3^); Fisher’s LSD (** *P* < 0.01). Bars represent group mean, error bars represent SEM, and dots represent individual scores

**
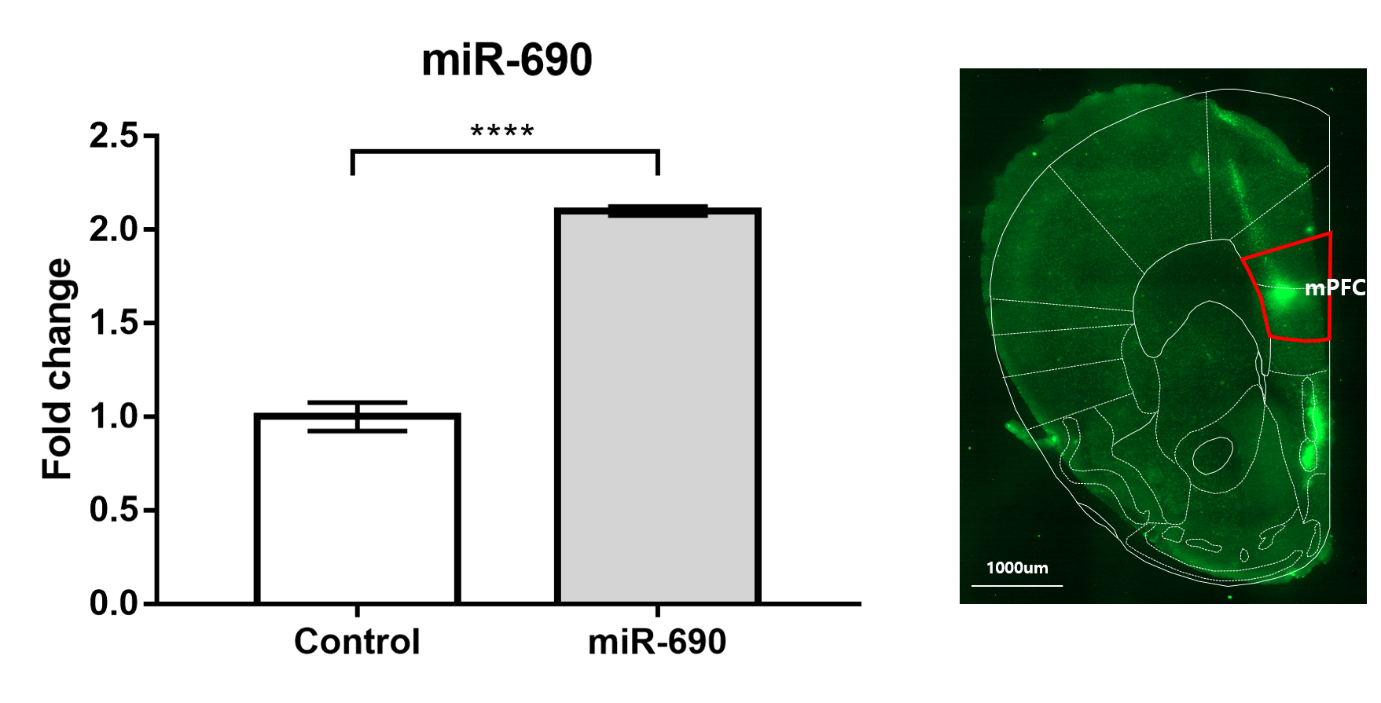
**

**Figure S3.** Expression level of miR-690 at D28 after introducing AAV-GFP-control and AAV-GFP-miR690, respectively, into the mPFC of mice. Ct values were normalized to that of internal controls (U6 and miR-16). Data are shown as means ± standard errors of the mean (SEM) (fold change) and analyzed by unpaired *t*-test (Control, n = 3; miR-690, n = 3). **** *P* < 0.0001. Image showing the location of the virus infusion site in the mPFC of the mouse brain (4×, Lionheart FX)


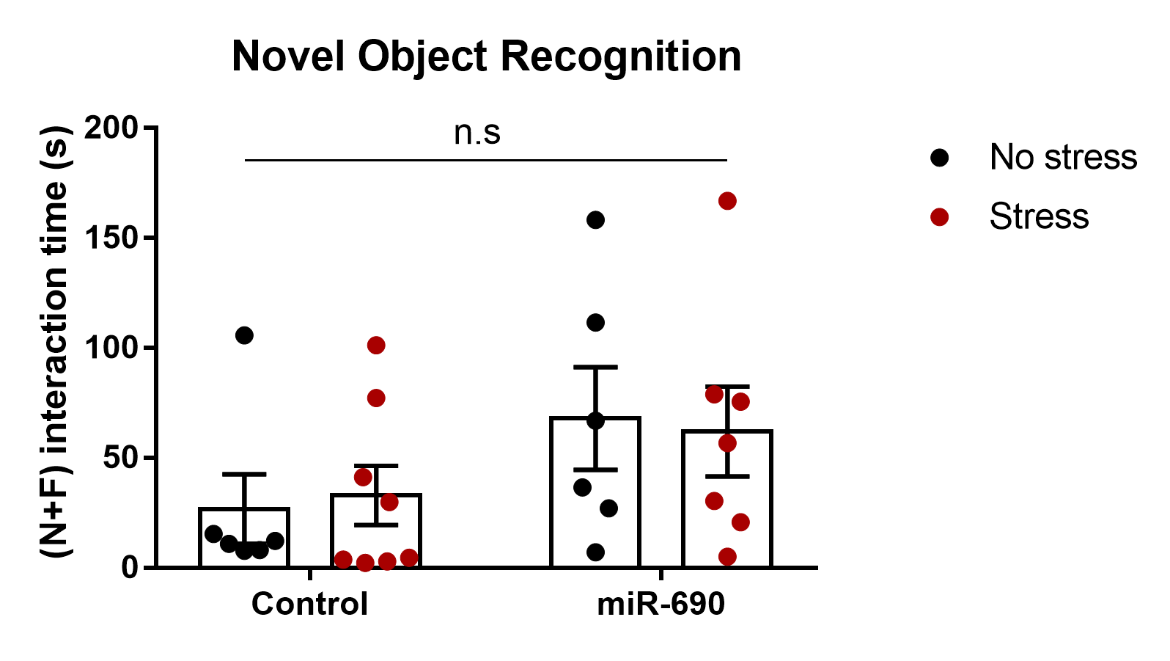


**Figure S4.** Novel and familiar objects interaction time in the novel object recognition test (NOR). Non-stressed control mice (n = 6); stressed control mice (n = 8); non-stressed miR-690 mice (n = 6); stressed miR-690 mice (n = 7). Data are analyzed by two-way ANOVA. Bars represent group mean and error bars represent SEM
